# Seroepidemiology of Neosporosis in Various Animals in the Qinghai-Tibetan Plateau

**DOI:** 10.3389/fvets.2022.953380

**Published:** 2022-07-19

**Authors:** Tongsheng Qi, Jingkai Ai, Jinfang Yang, Heng Zhu, Yuyu Zhou, Yulu Zhu, Heming Zhang, Qi Qin, Ming Kang, Yali Sun, Jixu Li

**Affiliations:** ^1^State Key Laboratory of Plateau Ecology and Agriculture, Qinghai University, Xining, China; ^2^Department of Veterinary Medicine, College of Agriculture and Animal Husbandry, Qinghai University, Xining, China; ^3^Qinghai Provincial Key Laboratory of Pathogen Diagnosis for Animal Diseases and Green Technical Research for Prevention and Control, Qinghai University, Xining, China

**Keywords:** *Neospora caninum*, IgG, IgM, animals, Qinghai-Tibetan Plateau, seroepidemiology

## Abstract

Neosporosis is a worldwide infectious disease caused by intracellular parasite *Neospora caninum* that is a major pathogen of abortion in cattle and neurological disorders in other hosts. However, limited data are available on animals exposed to *N. caninum* in the Qinghai-Tibetan Plateau Area (QTPA), and little is known about whether animals in the plateau area play an important role in the epidemiology of *N. caninum*. Therefore, indirect ELISAs based on a combination of *Nc*SAG1 and *Nc*GRA7 antigens were developed to examine both *N. caninum*-specific IgG and IgM antibodies in Tibetan sheep, yak, cow, pig, cattle, horse, chicken, camel, and donkey from the QTPA in this study. The results showed that all current species present- IgG and IgM-positive animals, and that the overall seroprevalence of *N. caninum* were 18.6 (703/3,782) and 48.1% (1,820/3,782) for the IgG and IgM antibodies, respectively. Further analysis found significant differences from different altitudes in IgG in Tibetan sheep and IgM in the yak. Hence, the present serological results indicate that the tested animal populations in the QTPA are suffering from *N. caninum* infections or have become carriers of *N. caninum* antibodies. To the best of our knowledge, this is the first report on current *N. caninum*-infected animals in the QTPA, the first epidemiology of neosporosis in cow and camel in China, and the first record of *N. caninum* IgM antibodies in all the surveyed animals in China. This study provides the latest valuable data on the epidemiology of neosporosis in China and in plateau areas of the world.

## Introduction

Neosporosis is a worldwide infectious disease caused by the obligate intracellular parasite protozoan *Neospora caninum*, which is a major pathogen of abortion in cattle and reproduction problems and neurological disorders in dogs (1–6). Canines are definitive hosts shedding oocysts in the environment that play an important role in the epidemiology of neosporosis associated with *N. caninum* infections in cattle and other intermediate hosts (e.g., sheep, pigs, goats, yaks, chickens, horses, and donkeys) (1, 3, 5).

*Neospora caninum* infection in a large spectrum of wild and domestic animals was described in many countries, especially cattle and dogs (1, 3, 5, 7–11). Although the prevalence of neosporosis in various animal hosts has been determined in several areas in China (12–18), limited data are available on domestic and wild animals exposed to *N. caninum* in the Qinghai-Tibetan Plateau Area (QTPA). For the epidemiology of neosporosis, serological ELISA diagnostic methods with highly specific and sensitive characteristics have been developed, and ELISAs based on specific antigens derived from *N. caninum*, especially for the case of surface antigen 1 (*Nc*SAG1) and dense granule protein 7 (*Nc*GRA7), were used to perform serological testing on parasitic infections in a large number of animal samples (19–23).

A variety of animals that are adapted to the high altitude and cold climate lives in the QTPA (24, 25), including Tibetan sheep (*Ovis aries*), yak (*Bos grunniens*), cow (*Bos taurus*), pig (*Sus domesticus*), cattle (*Bos taurus domestica*), horse (*Equus ferus caballus*), chicken (*Gallus gallusdomesticus*), camel (*Camelus bactrianus*), and donkey (*Equus asinus*). Diseases caused by infectious parasites have brought serious threats to the development of animal husbandries and human health. However, little is known whether the animals in this plateau area play an important role in the prevalence of *N. caninum*. Therefore, this present study aims to examine the serological prevalence of neosporosis using ELISAs based on the combination of recombinant SAG1 and GRA7 proteins in various animals in the QTPA. Our study should have major importance in epidemiological neosporosis in the plateau area.

## Materials and Methods

### Serum Samples

A total of 3,782 serum samples were collected in nine animal species from 2,000 m above sea level to 4,897 m in two cities and six prefectures of the QTPA with geographical coordinates of 31°36′-39°19′ N and 89°35′-103°04′ E from June 2021 to February 2022 (Table 1) including Tibetan sheep (*O. aries*), yak (*B. grunniens*), cow (*B. taurus*), pig (*S. domesticus*), cattle (*B. taurus domestica*), horse (*E. ferus caballus*), chicken (*G. gallusdomesticus*), camel (*C. bactrianus*), and donkey (*E. asinus*). Animal serum samples were frozen and stored at −20°C until assayed. All the procedures were carried out according to the ethical guidelines of Qinghai University.

**Table 1:** The sampling sites of animals in the QTPA in this study.

**State**	**Sampling site (altitude)**	**The number of collected and tested serum samples**									
		**Tibetan sheep**	**Yak**	**Cow**	**Pig**	**Cattle**	**Horse**	**Chicken**	**Camel**	**Donkey**	**Total**
HB	Menyuan (2,866 m)	36	20	0	0	11	0	0	0	0	67
	Gangcha (3,827 m)	404	0	0	0	0	14	0	0	0	418
	Haiyan (3,000 m)	145	104	0	0	0	10	0	0	0	259
	Total	585	124	0	0	11	24	0	0	0	744
HN	Gonghe (3,200 m)	190	20	0	0	0	265	0	0	0	475
	Guide (2,200 m)	0	0	0	30	0	0	0	0	0	30
	Total	190	20	0	30	0	265	0	0	0	505
HX	Delingha (2,980 m)	0	0	0	30	45	0	0	0	0	75
	Golmud (2,780 m)	0	20	0	20	0	0	0	0	0	40
	Tianjun (3,993 m)	8	0	0	0	0	0	0	0	0	8
	Wulan (4,000 m)	0	0	0	0	0	0	0	49	0	49
	Total	8	20	0	50	45	0	0	49	0	172
YS	Zhiduo (4,897 m)	0	20	0	0	0	0	0	0	0	20
GL	Maqin (4,100 m)	0	110	0	0	50	0	0	0	0	160
	Darlag (4,271 m)	0	86	0	0	0	0	0	0	0	86
	Banma (3,970 m)	0	133	0	0	0	0	0	0	0	133
	Total	0	329	0	0	50	0	0	0	0	399
HUN	Jianzha (2,063 m)	45	0	0	0	45	0	0	0	0	90
	Henan (4,200 m)	0	0	0	0	0	60	0	0	0	60
	Total	45	0	0	0	45	60	0	0	0	150
HD	Huzhu (2,535 m)	21	40	0	48	0	0	30	0	37	176
	Ledu (2,000 m)	0	20	389	67	100	0	0	0	0	576
	Minhe (2,174 m)	0	0	107	0	0	0	30	0	0	137
	Pingan (2,183 m)	53	0	0	77	0	0	0	0	0	130
	Total	74	60	496	192	100	0	60	0	37	1,019
XN	Datong (2,756 m)	0	219	0	24	200	30	80	0	0	553
	Huangzhong (2,645 m)	0	0	0	68	0	10	40	0	0	118
	Huangyuan (2,660 m)	0	0	0	48	0	0	30	0	0	78
	Xining (2,261 m)	0	0	0	44	0	0	0	0	0	44
	Total	0	219	0	184	200	40	150	0	0	793
Total		902	792	496	456	451	389	210	49	37	3,782

*HB, Haibei Tibetan Autonomous Prefecture, 36°44′-39°05′N and 98°5′-102°41′ E; HN, Hainan Tibetan Autonomous Prefecture, 34°38′-37°10′ N and 98°55–105°50′ E; HX, Haixi Mongolian and Tibetan Autonomous Prefecture, 35°01′-39°20′ N and 96°06′-99°42′ E; YS, Yushu Tibetan Autonomous Prefecture, 27°35′-36°35′ N and 89°35′-97°55′ E; GL, Guoluo Tibetan Autonomous Prefecture, 32°31′-35°37′ N and 96°54′-101°51′ E; HUN, Huangnan Tibetan Autonomous Prefecture, 34°04′-36°10′ N and 100°34′-102°28′ E; HD, Haidong city, 35°25.9′-37°05′ N and 100°41.5′-103°04′ E; XN, Xining city, 36°34′ N and 101°49′ E*.

### Expression and Purification of Recombinant *Nc*SAG1 and *Nc*GRA7 Proteins

The recombinant *Nc*SAG1 and *Nc*GRA7 were expressed and purified using the following protocols in this study: the SAG1 gene (GenBank: AF132217.1) and GRA7 gene (GenBank: JQ410455.1) were amplified by PCR from the cDNA of *N. caninum* parasites. Primers that included a BamH I site (underlined) in the forward primer 5′-CG *GGATCC* TCA GAA AAA TCA CCT CTA CT-3′, an EcoR I site (underlined) in the reverse primer 5′-CG *GAATTC* CGG ACC AAC ATT TTC AGC CGA CGA-3′ for *Nc*SAG1, a BamH I site (underlined) in the forward primer 5′-CG *GGATCC* GCT GGA GAC TTG GCA-3′ and an EcoR I site (underlined) in the reverse primer 5′-CG *GAATTC* CGC TAT TCG GTG TCT ACT TCC TG-3′ for *Nc*GRA7 were used. The PCR products digested with BamH I and EcoR I and inserted into the pGEX-6p-2 plasmid vector were treated with the same restriction enzymes (Roche, Switzerland). The IPTG was used to induce recombinant pGEX-6p-2-*Nc*SAG1 and pGEX-6p-2-*Nc*GRA7 expressions in *Escherichia coli* BL21 (DE3; New England BioLabs Inc., United States) at 37°C for 4 h, and then they were purified with the Glutathione Sepharose 4B beads (GE Healthcare Life Sciences) according to the manufacturer's instructions. The concentration of *Nc*SAG1 and *Nc*GRA7 proteins were measured with a bicinchoninic acid protein assay kit (Thermo Fisher Scientific, Inc., Rockford, IL, United States).

### Indirect ELISAs

IgG and IgM antibodies against *N. caninum* were detected by indirect ELISA tests based on the recombinant *Nc*SAG1 and *Nc*GRA7 proteins. The 1-μg/ml recombinant proteins measured with a bicinchoninic acid protein assay kit (Thermo Fisher Scientific, Inc., Rockford, IL, United States) were diluted in a coating buffer (0.05 M carbonate-bicarbonate, pH 9.6) to perform an indirect ELISA analysis: the current sera were diluted at 1:100, and the secondary antibodies of Rabbit Anti-Bovine IgM/HRP (bs-0327R-HRP; Bioss, China), Rabbit Anti-Bovine IgG H and L/HRP (bs-0326R-HRP; Bioss, China), Goat Anti-Horse IgM H and L/HRP (ab112879; Abcam, United Kingdom), Rabbit Anti-Horse IgG/HRP (bs-0308R-HRP; Bioss, China), Rabbit Anti-Sheep IgM/HRP (ab112763; Abcam, United Kingdom), Rabbit Anti-Sheep IgG H and L/HRP (AS023; Abclonal, China), Rabbit Anti-Pig IgG/HRP (bs-0309R-HRP; Bioss, China), HRP^*^Mab Pig IgM (Primadiagnostic, China), Goat Anti-Chicken IgG/HRP (bs-0310G-HRP; Bioss, China), Rabbit Anti-Chicken IgM/HRP (bs-0314R-HRP; Bioss, China), Goat Anti-Cow IgG H and L/HRP (ab102154; Abcam, United Kingdom), Sheep Anti-Cow IgM H and L/HRP (ab112752; Abcam, United Kingdom), Goat Anti-Donkey IgG H and L/HRP (ab6988; Abcam, United Kingdom), and Goat Anti-Camel IgG H and L/HRP (S003H; Nbbiolab, China) were diluted at 1:1,000−4,000. In this study, an ABTS [2,2'-azino-bis(3-ethylbenzothiazoline-6-sulfonic acid)] substrate was used to show the results at OD 415 nm. For the resulting judgment, the cut-off point was calculated as the mean value of OD 415 nm for standard *N. caninum*-negative sera kept in our laboratory (ten samples of each animal) plus three times the standard deviations of OD415 nm values of the negative controls: the mean *X* and standard deviation SD of the negative control results were calculated, and *X* + 3SD was the cut-off value of GRA7-ELISA and SAG1-ELISA. The OD 415 value was both greater than the respective cut-off values of GRA7-ELISA and SAG1-ELISA judged as positive, that is, the samples were judged as positive animals only when both SAG1-ELISA and GRA7-ELISA were positive. The positive and negative serum samples for neosporosis (gifts from Prof. Lijun Jia of Yanbian University, Jilin, China) were set as a control to confirm the indirect ELISAs.

### Statistical Analysis

To graph and analyze the data, the GraphPad Prism 8 software (GraphPad Software Inc., United States) was used. The prevalence and 95% confidence interval per pathogen species were calculated using the OpenEpi program (http://www.openepi.com/Proportion/Proportion.htm). A chi-squared test was conducted to compare the proportions of detected sample positivity in different regions and among different animals. Differences were considered to be statistically significant when resulting *P*-values were lower than 0.05.

## Results

### Cut-Off Values

To develop the epidemiology of neosporosis in current animals from the Qinghai-Tibetan Plateau, the cut-off values of indirect ELISA methods based on the two antigens, r*Nc*SAG1 and r*Nc*GRA7, were calculated to analyze both *N. caninum*-specific IgG and IgM antibodies in this study (Table 2).

**Table 2:** Cut-off values of current indirect ELISA tests in this study.

**Animal**	**Antigen**	**Antibody**	**Cut-off value**
Tibetan sheep	SAG1	IgG	0.373
		IgM	0.232
	GRA7	IgG	0.288
		IgM	0.254
Yak	SAG1	IgG	0.263
		IgM	0.137
	GRA7	IgG	0.234
		IgM	0.148
Cow	SAG1	IgG	0.273
		IgM	0.136
	GRA7	IgG	0.238
		IgM	0.149
Pig	SAG1	IgG	0.440
		IgM	0.120
	GRA7	IgG	0.376
		IgM	0.135
Cattle	SAG1	IgG	0.274
		IgM	0.149
	GRA7	IgG	0.248
		IgM	0.167
Horse	SAG1	IgG	0.262
		IgM	0.237
	GRA7	IgG	0.234
		IgM	0.401
Chicken	SAG1	IgG	0.397
		IgM	0.252
	GRA7	IgG	0.326
		IgM	0.145
Camel	SAG1	IgG	0.370
		IgM	–
	GRA7	IgG	0.288
		IgM	–
Donkey	SAG1	IgG	0.275
		IgM	–
	GRA7	IgG	0.268
		IgM	–

*–, not tested*.

### Indirect ELISAs

**Figure 1:**
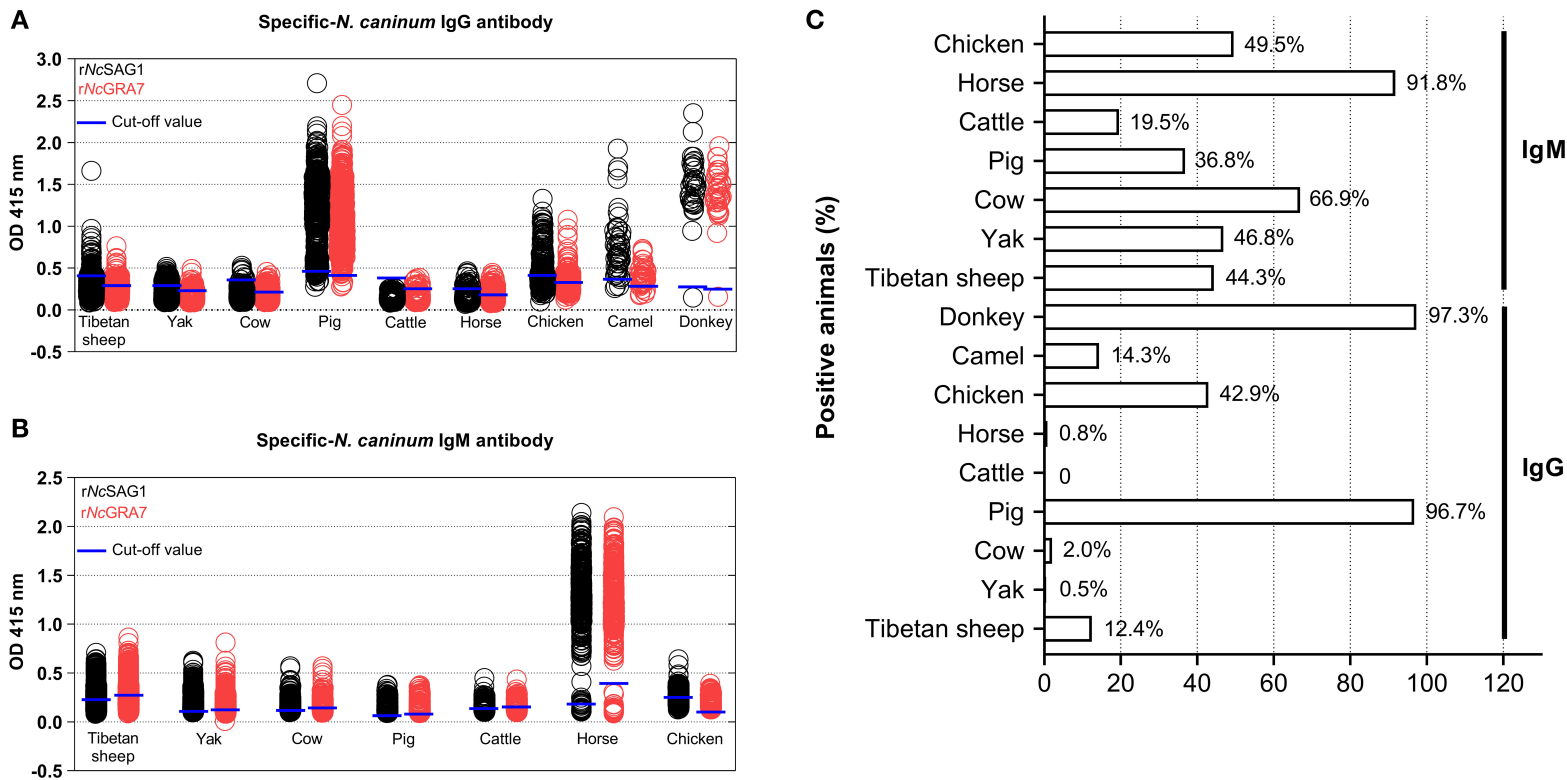
*Neospora caninum-*specific IgG and IgM antibodies in various animals in the Qinghai-Tibet Plateau Area were detected by indirect ELISA methods based on the *Nc*SAG1 and *Nc*GRA7 antigens in this study. The blue short lines represented the cut-off values. **(A)** *N. caninum*-specific-IgG antibodies, **(B)** *N. caninum*-specific IgM antibodies, and **(C)** positive animals for neosporosis (%).

Here, the combination of r*Nc*SAG1 and r*Nc*GRA7 proteins was used to examine the serological prevalence of neosporosis in various animals in the Qinghai-Tibetan Plateau. The overall seroprevalence of *N. caninum* in the examined animals was 18.6 (705/3,782) and 48.9% (1,850/3,782) for the IgG and IgM antibodies, respectively (Table 3 and Figure 1). Further analysis showed that out of the 3,782 animals, 330 (8.7%) were positive for both the IgG and IgM antibodies, and 2,275 (60.2%) were determined to be positive for at least one *N. caninum* indicator (Table 3). Moreover, the current study found that the 52% (469/902) of the Tibetan sheep, 46.7% (370/792) of the yaks, 67.3% (334/496) of the cows, 97.4% (444/456) of the pigs, 19.5% (88/451) of the cattle, 91.8% (357/389) of the horses, 61.9% (130/210) of the chickens, 85.7% (42/49) of the camels, and 97.3% (36/37) of the donkeys were positive for at least one parasitic indicator (IgG or IgM).

**Table 3:** Seroprevalence of *Neospora caninum-* specific IgG and IgM in animals in the QTPA.

**Animal**	**State**	**No**.	**Total IgG-positive (%, 95% CI)**	**Total IgM-positive (%, 95% CI)**	**Both IgG and IgM-positive (%, 95% CI)**	**Single-IgG-positive (%, 95% CI)**	**Single-IgM-positive (%, 95% CI)**
Tibetan sheep	HB	585	103 (17.6, 14.5–20.7)	362 (61.9, 57.9–65.8)	80 (13.7, 10.9–16.5)	23 (3.9, 2.4–5.5)	282 (48.2, 44.2–52.3)
	HN	190	2 (1.1, 0.4–2.5)	7 (3.7, 1.0–6.4)	1 (0.5, 0.5–1.6)	1 (0.5, 0.5–1.6)	6 (3.2, 0.7–5.6)
	HX	8	0	0	0	0	0
	HUN	45	7 (15.6, 5.0–26.1)	31 (68.9, 55.4–82.4)	7 (15.6, 5.0–26.1)	0	24 (53.3, 38.8–67.9)
	HD	74	0	0	0	0	0
	Total	902	112 (12.4, 10.3–14.6)	400 (44.3, 41.1–47.6)	88 (9.8, 7.8–11.7)	24 (2.7, 1.6–3.7)	312 (34.6, 31.5–37.7)
Yak	HB	124	0	24 (19.4, 12.4–26.3)	0	0	24 (19.4, 12.4–26.3)
	HN	20	0	0	0	0	0
	YS	20	0	4 (20.0, 2.5–37.5)	0	0	4 (20.0, 2.5–37.5)
	HX	20	0	0	0	0	0
	GL	329	2 (0.6, 0.2–1.4)	197 (59.9, 54.6–65.2)	2 (0.6, 0.2–1.4)	0	195 (59.3, 54.0–64.6)
	HD	60	2 (3.3, 1.2–7.9)	31 (51.7, 39.0–64.3)	1 (1.7, 1.6–4.9)	1 (1.7, 1.6–4.9)	30 (50.0, 37.3–62.7)
	XN	219	0	115 (52.5, 45.9–59.1)	0	0	115 (52.5, 45.9–59.1)
	Total	792	4 (0.5, 0.0–1.0)	371 (46.8, 43.4–50.3)	3 (0.4, 0.0–0.8)	1 (0.1, 0.1–0.4)	368 (46.5, 43.0–49.9)
Cow	HD	496	10 (2.0, 0.8–3.3)	332 (66.9, 628–71.1)	7 (1.4, 0.4–2.4)	3 (0.6, 0.1–1.3)	325 (65.5, 61.3–69.7)
	Total	496	10 (2.0, 0.8–3.3)	332 (66.9, 628–71.1)	7 (1.4, 0.4–2.4)	3 (0.6, 0.1–1.3)	325 (65.5, 61.3–69.7)
Pig	HN	30	25 (83.3, 70.0–96.7)	6 (20.0, 5.7–34.3)	6 (20.0, 5.7–34.3)	19 (63.3, 46.1–80.6)	0
	HX	50	50 (100, 100–100.0)	25 (50.0, 36.1–63.9)	25 (50.0, 36.1–63.9)	25 (50.0, 36.1–63.9)	0
	HD	192	190 (99.0, 97.5–100.4)	62 (32.3, 25.7–38.9)	62 (32.3, 25.7–38.9)	128 (66.7, 60.0–73.3)	0
	XN	184	176 (95.7, 92.7–98.6)	75 (40.8, 33.7–47.9)	73 (39.7, 32.6–46.7)	103 (56.0, 48.8–63.2)	2 (1.1, 0.4–2.6)
	Total	456	441 (96.7, 95.1–98.3)	168 (36.8, 42.4–41.3)	166 (36.4, 32.0–40.8)	275 (60.3, 55.8–64.8)	2 (0.4, 0.2–1.0)
Cattle	HB	11	0	3 (27.3, 1.0–53.6)	0	0	3 (27.3, 1.0–53.6)
	HX	45	0	4 (8.9, 0.6–17.2)	0	0	4 (8.9, 0.6–17.2)
	HUN	45	0	23(51.1, 36.5–65.7)	0	0	23(51.1, 36.5–65.7)
	GL	50	0	10 (20.0, 8.9–31.1)	0	0	10 (20.0, 8.9–31.1)
	HD	100	0	25 (25.0, 16.5–33.5)	0	0	25 (25.0, 16.5–33.5)
	XN	200	0	23(11.5, 7.1–15.9)	0	0	23(11.5, 7.1–15.9)
	Total	451	0	88 (19.5, 15.9–23.2)	0	0	88 (19.5, 15.9–23.2)
Horse	HB	24	0	24 (100, 100.0–100.0)	0	0	24 (100, 100.0–100.0)
	HN	265	1 (0.4, 0.4–1.1)	235 (88.7, 84.9–92.5)	1 (0.4, 0.4–1.1)	0	234 (88.3, 84.4–92.2)
	HUN	60	2 (3.3, 1.2–7.9)	59 (98.3, 95.1–101.6)	2 (3.3, 1.2–7.9)	0	57 (95.0, 89.5–100.5)
	XN	40	0	39 (97.5, 92.7–102.3)	0	0	39 (97.5, 92.7–102.3)
	Total	389	3 (0.8, 0.1–1.6)	357 (91.8, 89.0–94.5)	3 (0.8, 0.1–1.6)	0	354 (91.0, 88.2–93.8)
Chicken	HD	60	36 (60.0, 47.6–72.4)	31 (51.7, 39.0–64.3)	29 (48.3, 35.7–61.0)	7 (11.7, 3.5–19.8)	2 (33.0, 1.2–7.9)
	XN	150	54 (36.0, 28.3–43.7)	73 (48.7, 40.7–56.7)	34 (22.7, 16.0–29.4)	20 (13.3, 7.9–18.8)	39 (26.0, 19.0–33.0)
	Total	210	90 (42.9, 36.2–49.6)	104 (49.5, 42.8–56.3)	63 (30.0, 23.8–36.2)	27 (12.9, 8.3–17.4)	41 (19.5, 14.2–24.9)
Camel	HX	49	7 (14.3, 4.5–24.1)	–	–	7 (14.3, 4.5–24.1)	–
	Total	49	7 (14.3, 4.5–24.1)	–	–	7 (14.3, 4.5–24.1)	–
Donkey	HD	37	36 (97.3, 92.1–102.5)	–	–	36 (97.3, 92.1–102.5)	–
	Total	37	36 (97.3, 92.1–102.5)	–	–	36 (97.3, 92.1–102.5)	–
Total		3,782	703 (18.6, 17.3–19.8)	1,820 (48.1, 46.5–49.7)	330 (8.7, 7.8–9.6)	373 (9.9, 8.9–10.8)	1,490 (39.4, 37.8–41.0)

*No., No. of tested in this study; %, prevalence; 95% CI, 95% confidence interval; HB, Haibei Tibetan Autonomous Prefecture; HN, Hainan Tibetan Autonomous Prefecture; HX, Haixi Mongolian and Tibetan Autonomous Prefecture; YS, Yushu Tibetan Autonomous Prefecture; GL, Guoluo Tibetan Autonomous Prefecture; HUN, Huangnan Tibetan Autonomous Prefecture; HD, Haidong city; XN, Xining city*.

As shown in Table 3 and Figure 1, to analyze the positive animals found that the donkey was the most prevalent animal (97.3%, 36/37) for IgG positivity, followed by pig (96.7%, 441/456), chicken (42.9%, 90/210), camel (14.3, 7/49), Tibetan sheep (12.4%, 112/902), cow (2.0%, 10/496), horse (0.8, 3/389), yak (0.5%, 4/792), and cattle (0, 0/451). While analysis for the IgM antibody positivity, the horse was the most prevalent animal (91.8%, 357/389) for IgM positivity, followed by cow (66.9%, 332/496), chicken (49.5%, 104/210), yak (46.8%, 371/792), Tibetan sheep (44.3%, 400/902), pig (36.8%, 168/456), and cattle (19.5, 88/451).

### Analysis of Influence of Altitude on Seroprevalence of *N. caninum*

To analyze the influence of seroprevalence from different heights above sea levels in the sampling areas, all the animals were differentiated into three groups, namely the 2,000–3,000-, 3,000–4,000-, and 4,000–5,000-m altitude groups (Table 4). The analysis found significant differences (*P* < 0.05) from different altitudes in *N. caninum* specific-IgG in the Tibetan sheep, and IgM in the yak, but there was no difference in other current animals.

**Table 4:** Analysis of the influence of altitude on the seroprevalence of the *Neospora caninum* IgG and IgM antibodies and distribution of toxoplasmosis and neosporosis in the QTPA.

**Animal**	**Antibody**	**2,000–3,000 m**		**3,000–4,000 m**		**4,000–5,000 m**		***P*-value**
		**Tested**	**Positive (%)**	**Tested**	**Positive (%)**	**Tested**	**Positive (%)**	
Tibetan sheep	IgG	155	11 (7.1)	747	103 (13.8)	0	–	0.0403
	IgM		67 (43.2)		363 (48.6)		–	0.4631
Yak	IgG	319	2 (0.6)	257	0	216	2 (0.9)	0.3427
	IgM		166 (52.0)		50 (19.5)		155 (71.8)	<0.0001
Cow	IgG	496	10 (2.0)	0	–	0	–	–
	IgM		332 (66.9)		–		–	–
Pig	IgG	456	441 (96.7)	0	–	0	–	–
	IgM		168 (36.8)		–		–	–
Cattle	IgG	401	0	0	–	50	0	–
	IgM		78 (19.5)		–		10 (20.0)	0.9397
Horse	IgG	40	0	289	1 (0.3)	60	2 (3.3)	0.0517
	IgM		39 (97.5)		259 (89.6)		59 (98.3)	0.8645
Chicken	IgG	210	90 (42.9)	0	–	0	–	–
	IgM		104 (49.5)		–		–	–
Camel	IgG	0	–	0	–	49	7 (14.3)	–
Donkey	IgG	37	36 (97.3)	0	–	0	–	–

*–, not tested; %, prevalence; n, no significant difference*.

## Discussion

The SAG1 and GRA7 of *N. caninum* have been identified and tested as important candidates for serological diagnosis of neosporosis in animals (19–23). To develop the epidemiology of neosporosis in current animals from the Qinghai-Tibetan Plateau, indirect ELISA methods based on the two antigens were established to detect both *N. caninum*-specific IgG and IgM antibodies in this study. Although the SAG1 and GRA7 antigens of *N. caninum* were identified to be expressed in different stages of parasitic life cycles (19–23), a combination of recombinant protein-based ELISAs offers the best evidence for the diagnosis of *N. caninum* infection in this study. This study was the first to combine the SAG1 and GRA7 proteins in detection of *N. caninum*, and current ELISAs based on them confirmed that seropositive animals for neosporosis were present in the examined sampling areas. The results above showed that the animals in the QTPA present *N. caninum* infections, suggesting that the animals could have key roles in the transmission and prevalence of *N. caninum* in the plateau area. To the best of our knowledge, this is the first report of *N. caninum* infection in the present animals in the QTPA, the first epidemiology of neosporosis in the cow and camel in China, and the first record of *N. caninum* IgM antibodies in all the surveyed animals in China.

A variety of unique animals has been domesticated in the QTPA, which is the largest plateau with the highest average altitude on the planet, and the animals share water and food in the plateau area (24, 25). Infectious diseases caused by *N. caninum* parasites are common in animals in the world (1, 3, 5). Of the investigated animal species in this study, the IgG positivity was from 0 (cattle) to 97.3% (donkey), while that of IgM was from 19.5 (cattle) to 91.8% (horse). These findings showed the higher epidemiology of neosporosis in current animals in the QTPA compared with the 5.14, 8.4, 1.9, 23.1, and 9.5% IgG seroprevalence in yaks, Tibetan sheep, pigs, chickens, and equines in several provinces in China (12–18). Moreover, the *N. caninum* positivity rates of the present animals were also higher than the 13.46% in equines in the world (17), 40% in pigs in Italy (26), and 3.9% in camels in Iran (27). Interestingly, the current study found that 97.3% of the donkeys (36/37) and 96.7% of the pigs (441/456) were IgG-positive. Intermediate hosts probably become infected with *N. caninum* mainly through ingestion of foods or drinking water contaminated by sporulated *N. caninum* oocysts (1–3). Most of the pig samples in this study were collected from Xining and Haidong cities, and the sampling areas seem to present a large number of important risk factors such as stray dogs for *N. caninum* infection (28). Moreover, the current pig farms in the Qinghai-Tibet Plateau are non-intensive farming, which means more convenient conditions for *N. caninum* oocysts seeded by the dogs are exposed to pigs. For donkeys, they were likely exposed to foods and water contaminated with oocysts in Haidong city, while the limitation of this study is that the sample size is too small for donkeys. On the other hand, although *N. caninum* is a major pathogen of abortion and reproduction problems in cattle and dogs (1–3), its infection in various animal species including the current animals has been reported (7–18). However, this study found higher IgM positivity rates in the horses (91.8%) and chickens (49.5%) than in the yaks and cattle. Actually, the DNAs of *N. caninum* have been molecularly amplified in horses and chickens (12, 17, 29), and it is possible that the current detection of high IgM positivity rate may indicate the possibility that neosporosis is developing, but this must be combined with clinical observations and isolation of *N. caninum* parasites to confirm. The present serological results indicate that the Qinghai-Tibet Plateau is also an endemic area of *N. caninum*, which may be related to the existence of a large number of stray dogs and herding sheepdogs there.

Generally considered the IgG antibodies rise to protective levels after infection and remain detectable for years while the lower occurrence of IgM antibodies is within days to a couple of weeks, moreover, *N. caninum* positive for IgG + IgM are proposed to be chronic reactivated cases. The current study found that the seroprevalence of the *N. caninum* IgM antibody in Tibetan sheep, yaks, cows, horses, cattle, and chickens was higher than that of the IgG antibody, but in pigs IgG was lower. Moreover, 36.4% (166/456) of the pigs and 30% (63/210) of the chickens were tested to be both IgG- and IgM-positive. Furthermore, the current results found low IgG positivity rates present in the cattle (0), yaks (0.5%) and cows (2.0%) but more than 19.5% IgM positivity rates in the animals. Therefore, the present study may suggest the prevalence of acute neosporosis in Tibetan sheep, yaks, cows and horses, chronic re-emergence of neosporosis in pigs and chickens, and chronic neosporosis in cattle in the testing area. These reveal that the animal populations are suffering from *N. caninum* infection or have become carriers of *N. caninum* antibodies after the infection.

To show the influence on seroprevalence from different heights above sea levels of the sampling areas in the 2,000–3,000, 3,000–4,000, and 4,000–5,000 m altitudes in this study, an analysis was conducted. It was found that there were only significant differences (*P* < 0.05) from different altitudes in *N. caninum*-specific-IgG in Tibetan sheep and IgM in yak, but that there was no difference in other current animals. Nonetheless, because of the limitation of sampling sites and the number of samples, it cannot be said that altitude is a key factor affecting the prevalence of *N. caninum* in a strict sense. But the differences present may be because of the different feeding methods for various animals: the animals are grazing in the high-altitude areas sharing the common waters and foods, while a large number of humans and the definitive-host dogs especially stray dogs are activating in the low altitude areas leading to these food-borne animals have frequently exposed the infecting source and cause *N. caninum* infections.

In conclusion, this study is the first to demonstrate *N. caninum* infections using serological ELISAs based on the combination of recombinant SAG1 and GRA7 proteins in various animals in the current plateau area, and determination of the *N. caninum* IgM antibody in these animals in China. These give latest valuable data on the epidemiology of neosporosis in China and in plateau areas of the world. Future studies should focus on clinical cases of neosporosis such as abortions and neurological disorders in animals and perform isolation of *N. caninum* parasites in domestic and wild animals including definitive and intermediate hosts in the Qinghai-Tibetan Plateau Area.

## Data Availability Statement

The original contributions presented in the study are included in the article/supplementary material, further inquiries can be directed to the corresponding author.

## Ethics Statement

The animal study was reviewed and approved by Ethics Committee of Qinghai University (No. SL-2021016, date of approval: March 16, 2021). Written informed consent was obtained from the owners for the participation of their animals in this study.

## Author Contributions

TQ: conceptualization, data curation, formal analysis, investigation, writing (original draft), and writing (review and editing). JA and JY: data curation, formal analysis, and investigation. HZ: investigation, formal analysis, and validation. YYZ, YLZ, HMZ, and QQ: data curation and formal analysis. MK and YS: resources and writing (review and editing). JL: conceptualization, funding acquisition, resources, writing (original draft), and writing (review and editing). All authors contributed to the article and approved the submitted version.

## Funding

This study was supported by the Natural Science Foundation of Qinghai Province of China [Grant Nos: 2022-ZJ-956Q and 2022-ZJ-940Q] and the Veterinary Bureau Scientific Research Foundation of Qinghai Province [Grant No: NMSY-2021-05].

## Conflict of Interest

The authors declare that the research was conducted in the absence of any commercial or financial relationships that could be construed as a potential conflict of interest.

## Publisher's Note

All claims expressed in this article are solely those of the authors and do not necessarily represent those of their affiliated organizations, or those of the publisher, the editors and the reviewers. Any product that may be evaluated in this article, or claim that may be made by its manufacturer, is not guaranteed or endorsed by the publisher.
